# Overexpression of *CfICE1* from *Cryptomeria fortunei* Enhances Cold, Drought and Salt Stress in Poplar

**DOI:** 10.3390/ijms232315214

**Published:** 2022-12-02

**Authors:** Lijuan Zhu, Junjie Yang, Yingting Zhang, Hailiang Hu, Jiebing Cui, Jinyu Xue, Jin Xu

**Affiliations:** Key Laboratory of Forest Genetics & Biotechnology of Ministry of Education, Co-Innovation Center for Sustainable Forestry in Southern China, College of Forestry, Nanjing Forestry University, Nanjing 210037, China

**Keywords:** *CfICE1*, cold resistance, drought resistance, salt resistance, *Cryptomeria fortune*, poplar (*Populus davidiana × P. bolleana*)

## Abstract

*ICE1*, a regulator of the cold-inducible transcriptome and freezing tolerance, is currently widely believed to be involved in plant resistance to cold stress. In this study, *CfICE1* from *Cryptomeria fortunei* was transformed into poplar. Physiological indicators of transgenic, empty vector and wild-type poplar after abiotic stress (cold, drought and salt) were determined. Transgenic lines had a higher chlorophyll content, antioxidant enzyme activity and soluble protein content, as well as a lower malondialdehyde and hydrogen peroxide content. The ultrastructure of the plant was observed by transmission electron microscopy, and after stress, the cell structure of the transgenic line was more complete than that of the wild type. *CfICE1* was upregulated in transgenic poplar trees after abiotic stress (cold, drought and salt). The *CfICE1* transgenic plants improved plant resistance by regulating the *CBF* gene of poplar under cold and salt stress. In terms of plant responses to abiotic stress, this study showed that overexpression of *CfICE1* improved the cold, drought and salt tolerance of poplars.

## 1. Introduction

The growth and development of plants depends to a great extent on their environment. Salt, drought and cold are common stress conditions that adversely affect plant growth [1,2]. Plants that grow under certain stress conditions not only have the ability to resist that stress but also have the ability to resist other stresses via a phenomenon called cross adaptation [3,4]. Some cold-resistant transgenic plants also improved their drought resistance. In plants, when cold resistance increased, cell dehydration was reduced, thus increasing the drought tolerance of the plant. Thus, the molecular mechanisms underlying the reaction to cold and drought conditions were very similar [5]. Salt, drought, and cold induced the accumulation of reactive oxygen species [6], which are disruptors of plant stress damage and signals that induce reactive oxygen species scavengers and other protective mechanisms [7,8]. Understanding the characteristics of plant physiological and biochemical responses under abiotic stress is an important indicator for evaluating the effect of plant resistance to abiotic stress [9,10].

To adapt to various stresses, plants have evolved corresponding regulatory pathways. Transcription factors promote the function of stress resistance gene resources by enhancing the action of some key regulators and fundamentally improving the stress resistance of plants [11,12]. It is well known that the *C-repeat-binding factor* (*CBF*) gene plays a key role in plant tolerance to abiotic stresses, such as cold, drought and salt [13,14]. Previous studies have shown that *CBF1*, a member of the *CBF* gene family, can activate the expression of most subsets of downstream cold-regulated (*COR*) genes in cold and salt environments, thereby protecting plants from harm [15]. The expression of *CBF* is regulated by genes, such as *ICE*. The *ICE–CBF–COR* conduction pathway is a well-established transcriptional regulatory pathway related to cold adaptation. *ICE* specifically binds to the promoter sequence of *CBF* under cold stress to induce the expression of *CBF*, and then *CBF* binds to the DRE sequence of the downstream target gene promoter to induce the expression of *COR*, thereby improving the frost resistance of the plant [16]. Overexpression of *ICE1*, a master switch that controls the expression levels of *CBF* and its downstream components in the signal transduction pathway, could improve plant stress resistance in transgenic plants. A study showed that *CBF3*, the main target of *ICE1*, played a role in the early response to drought stress at flowering, suggesting that *ICE1* was resistant to water-associated stress [17]. To explore the potential function of *ICE1* in *Poncirus trifoliata*, some researchers observed changes in the expression of *PtrICE1* under abiotic stress and found that the expression of *PtrICE1* was upregulated under cold, drought and salt stress [18].

In a previous study [19], we cloned *CfICE1* from *C. fortunei*., which has a length of 575 aa and encodes a protein sequence that contains a serine-rich motif, a basic helical-ring-helical zipper (bHLH-Zip), an ACT domain, and a nuclear localization signal (NLS). In addition, *CfICE1* was transformed into *Arabidopsis thaliana* to obtain transgenic seedlings, and after low-temperature treatment, it was found that *CfICE1* overexpression could improve the antioxidant enzyme activity of the plants, reduce the malondialdehyde content in the plants, and endow transgenic plants with good cold resistance. However, these early works were carried out on herbaceous plants. The role of *CfICE1* in the cold resistance of perennial woody plants needs to be further verified. In addition to the synergistic nature of stresses, such as cold, drought and salt, the role that *ICE1*, the main switch controlling the expression levels of components downstream of *CBF* and its signal transduction pathways, plays when plants are under abiotic stress remains to be studied. Therefore, to further verify the function of the *CfICE1* gene, we investigated whether *CfICE1* was involved in the regulation of salt stress and drought stress tolerance based on analyses of transgenic woody plants.

## 2. Results

### 2.1. CfICE1 Overexpression Caused Morphological Changes in Transgenic Poplars

**Figure 1:**
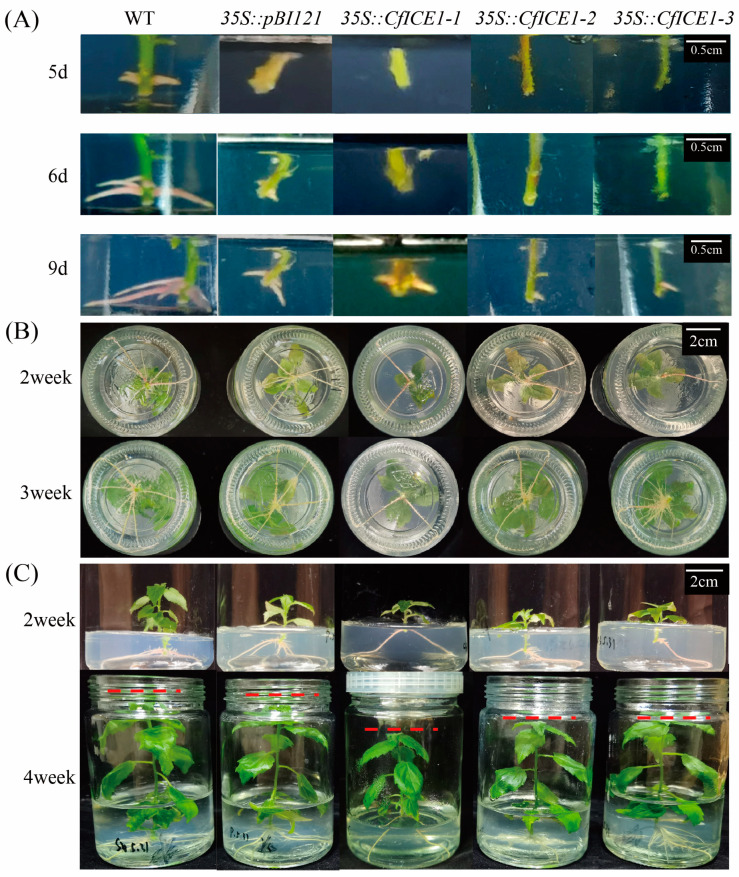
Rooting process of transgenic, empty vector and wild-type poplar lines. (**A**) The formation process of poplar adventitious roots. (**B**) Top view of poplar adventitious roots. (**C**) Comparison of plant height among transgenic, empty vector and wild-type poplar lines.

To reveal the function of *CfICE1* in woody plants, we transferred *CfICE1* into poplar, and after obtaining transgenic seedlings, we selected plants with high expression and significant differences from WT for further analysis. Figure S1 shows the gene expression in each plant after treatment at 4 °C. The expression level of the target gene was 7726.47-, 14398.09- and 9240.01-fold in WT plants in the *35S::CfICE1-1*, *35S::CfICE1-2* and *35S::CfICE1-3* lines, respectively. The expression level of the target gene was similar between *35S::pBI121* and WT. Observations of the rooting process of WT and transgenic plants showed that the adventitious roots of the transgenic plants formed later than those of the WT (Figure 1A) and were less developed (Figure 1B). Then, the 4-week-old transgenic, empty vector and WT poplar tissue culture seedlings were transplanted into plastic pots filled with soil and placed in a controlled environment room for 1 month; their plant heights and the phenotypes of the fourth unfolded leaf were compared. It was found that the WT was significantly superior to transgenic lines regarding plant height (Figure 1C). In addition, the leaf shape was similar between WT and transgenic plants, and there was no significant difference in the aspect ratio (Table 1). Briefly, overexpression of *CfICE1* in poplar slightly delayed adventitious root formation and ultimately affected the height of transgenic plants.

### 2.2. Physiological Response of CfICE1 Transgenic Poplar Plants to Cold Stress

**Figure 2:**
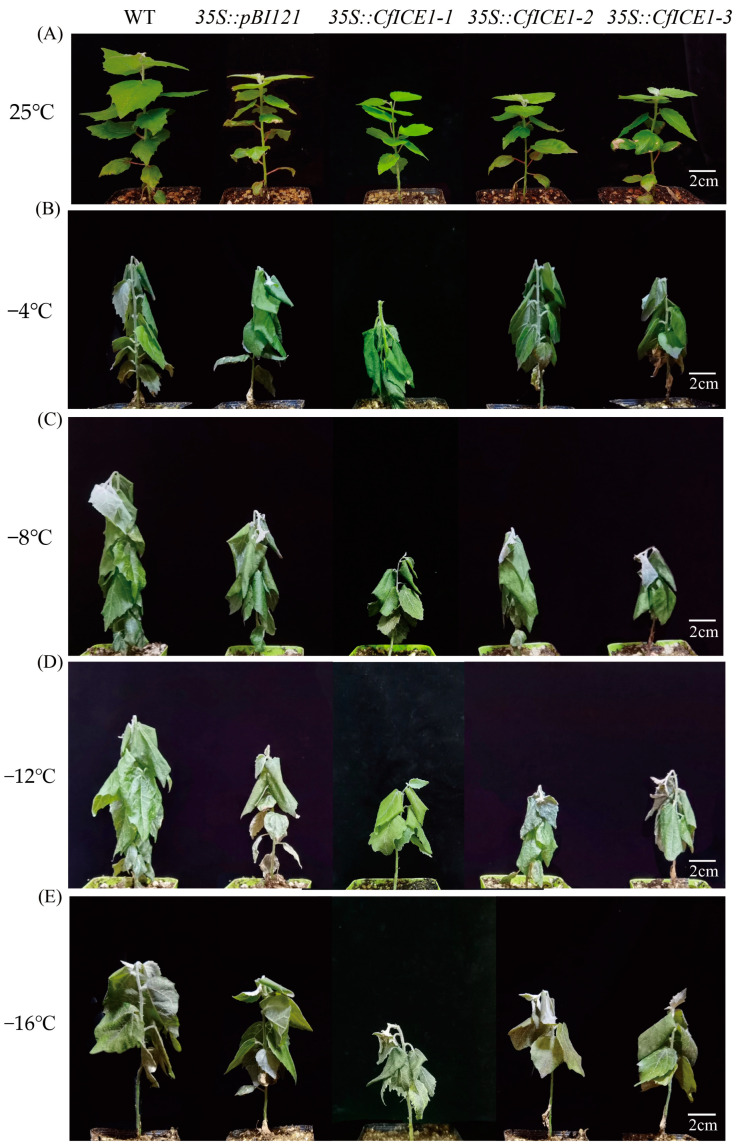
Phenotypic changes of transgenic, empty vector and wild-type poplar lines under cold stress at (**A**) 25 °C, (**B**) −4 °C, (**C**) −8 °C, (**D**) −12 °C and (**E**) −16 °C.

**Figure 3:**
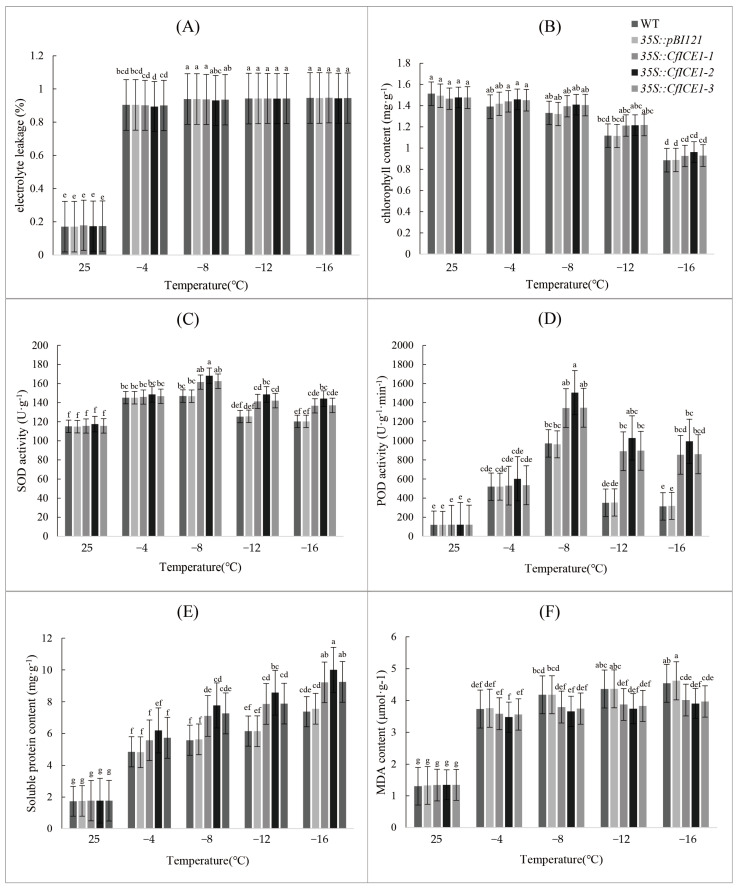
Physiological indices of transgenic, empty vector and wild-type poplar lines under cold stress: (**A**) electrolyte leakage, (**B**) chlorophyll content, (**C**) SOD activity, (**D**) POD activity, (**E**) soluble protein content, (**F**) MDA content. The abscissas of all the above physiological indicators are the treatment temperature. Each bar indicates the average ± SD (n = 3), and lowercase letters above each bar indicate significant differences (*p* ≤ 0.05).

To verify the role of *CfICE1* in hardiness, we cold treated *35S::CfICE1-1*, *35S::CfICE1-2*, *35S::CfICE1-3*, *35S::pBI121* and WT plants at different temperatures. After cold treatment, the five plants all showed wilted leaves and droopy stems (Figure 2). The electrolyte leakage (EL) of plant leaves can be used to characterize the damage to the plant cell membrane [20]. Under the low-temperature treatment, the EL was more than 90% (Figure 3A), indicating that both the WT and transgenic plants had suffered severe freezing damage and that the cell membrane had been greatly damaged. The plant chlorophyll content decreased with decreasing temperature (Figure 3B). Under nonstress conditions, there was no significant difference in enzyme activity (SOD and POD) between transgenic and WT plants; however, as the temperature decreased, there were differences in enzyme activity between lines (Figure 3C,D). Under −8 °C treatment, the SOD activity of both WT and transgenic plants was maximized. Under the same cold treatment, the SOD activity between WT and *35S::pBI121* was similar, while the SOD activity of *35S::CfICE1* was higher than that of WT, which indicated that the pBI121 vector had no effect in the transgenic plants and that *CfICE1* could increase the SOD activity of plants under cold stress. After treatment at −16 °C, the SOD activity of *35S::CfICE1-2* was 1.2 times that of WT. The trend of POD activity with decreasing temperature stress was consistent with that of SOD. The overall POD activity decreased rapidly from −8 to −12 °C and decreased slowly from −12 to −16 °C. The POD activity of *35S::CfICE1-2* decreased by 1.51 times between −8 °C and −16 °C, while that of WT was 3.1 times lower. After treatment at −16 °C, the POD activity of *35S::CfICE1-2* was 3.18 times that of WT. With the decrease in temperature, the soluble protein content and malondialdehyde (MDA) content of each plant showed an increasing trend. However, under the same cold treatment, the soluble protein content of *35S::CfICE1* was higher than that of WT (Figure 3E), while the MDA content of *35S::CfICE1* was lower than that of WT (Figure 3F). These physiological indicators suggested that transgenic *CfICE1* could improve the cold resistance of poplar.

### 2.3. Physiological Response of CfICE1 Transgenic Poplar Plants to Salt Stress

**Figure 4:**
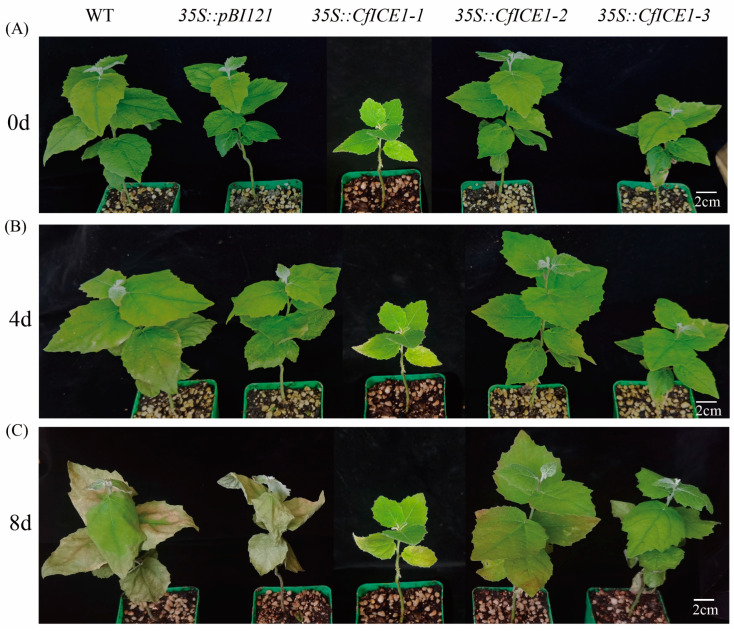
Phenotypic changes of transgenic, empty vector and wild-type poplar lines under (**A**) 0, (**B**) 4 and (**C**) 8 days of salt stress.

**Figure 5:**
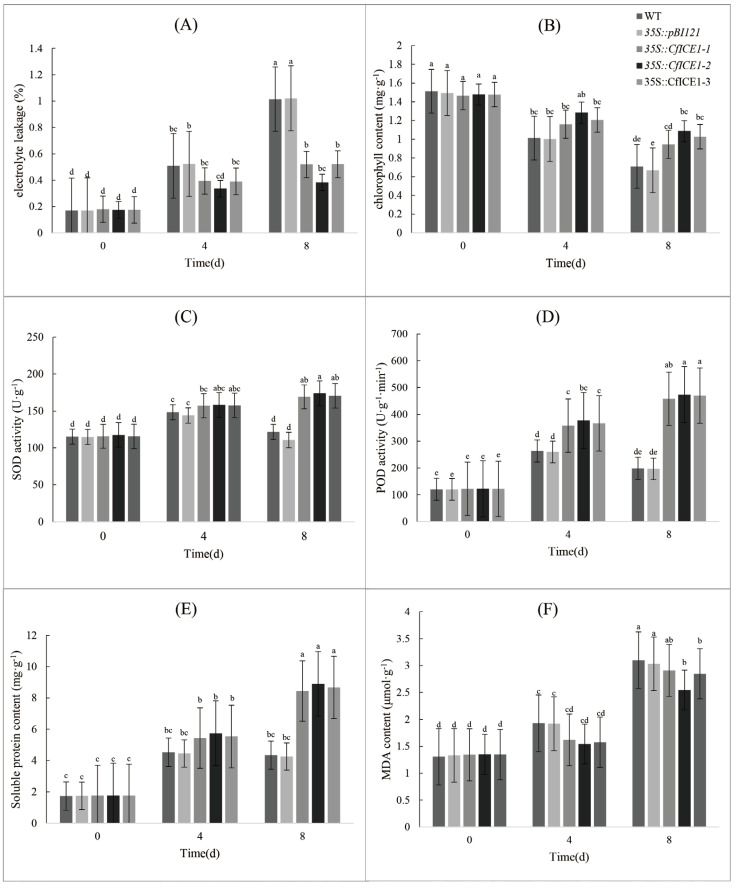
Physiological indices of transgenic, empty vector and wild-type poplar lines under salt stress: (**A**) electrolyte leakage, (**B**) chlorophyll content, (**C**) SOD activity, (**D**) POD activity, (**E**) soluble protein content, (**F**) MDA content. The abscissas of all the above physiological indicators are the days of salt treatment. Each bar indicates the average ± SD (n = 3), and lowercase letters above each bar indicate significant differences (*p* ≤ 0.05).

To investigate whether overexpression of *CfICE1* improves salt tolerance in plants, we also treated these five plant lines with salt (Figure 4). Unlike after cold treatment, there were visible differences in the phenotype between WT and transgenic poplars after salt treatment. After 4 days of salt treatment, the true leaves below the fifth leaf of WT became thin and wrinkled, and the periphery turned white–yellow (Figure 4B); after 8 days of salt treatment, WT and *35S::pBI121* leaves were obviously yellowed while *35S::CfICE1-1*, *35S::CfICE1-2* and *35S::CfICE1-3* leaves remained green (Figure 4C). This phenomenon was confirmed by the EL and chlorophyll content (Figure 5A,B). When treated with salt for 8 days, the chlorophyll content of WT and *35S::pBI121* was significantly reduced compared to that of the transgenic lines, and the EL was as high as 100%. In the antioxidant enzyme reaction, the enzyme activities (SOD and POD) of transgenic plants continued to increase, while the enzyme activities of WT plants treated with salt for 8 days were much lower than those of salt-treated plants for 4 days (Figure 5C,D). The soluble protein that protects important substances and biofilms of cells showed no significant difference after 4 and 8 days of salt treatment in WT plants. This might be because when the stress reaches a certain level, protein synthesis is inhibited and even causes protein inactivation. The soluble protein of transgenic plants increased significantly, indicating that the transgenic *CfICE1* gene could improve the salt tolerance of poplar (Figure 5E). The MDA content of transgenic plants was lower than that of WT, especially after 8 days of salt stress (Figure 5F).

### 2.4. Physiological Response of CfICE1 Transgenic Poplar Plants to Drought Stress

**Figure 6:**
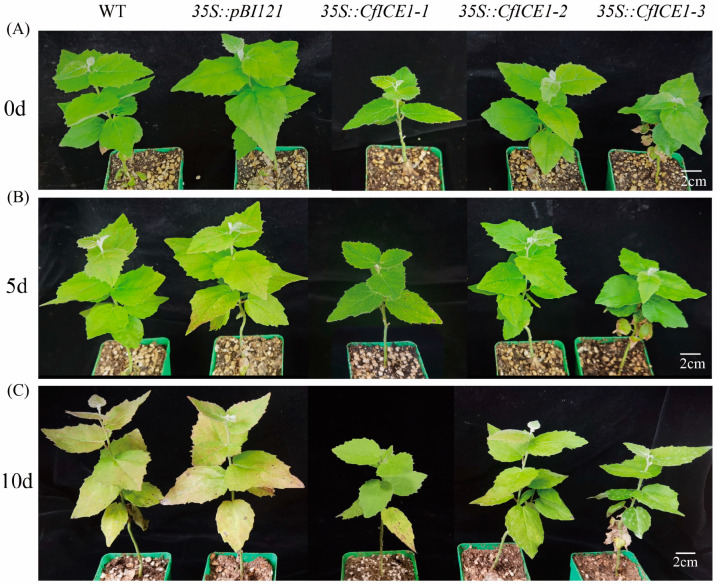
Phenotypic changes of transgenic, empty vector and wild-type poplar lines under (**A**) 0, (**B**) 5 and (**C**) 10 days of drought stress.

**Figure 7:**
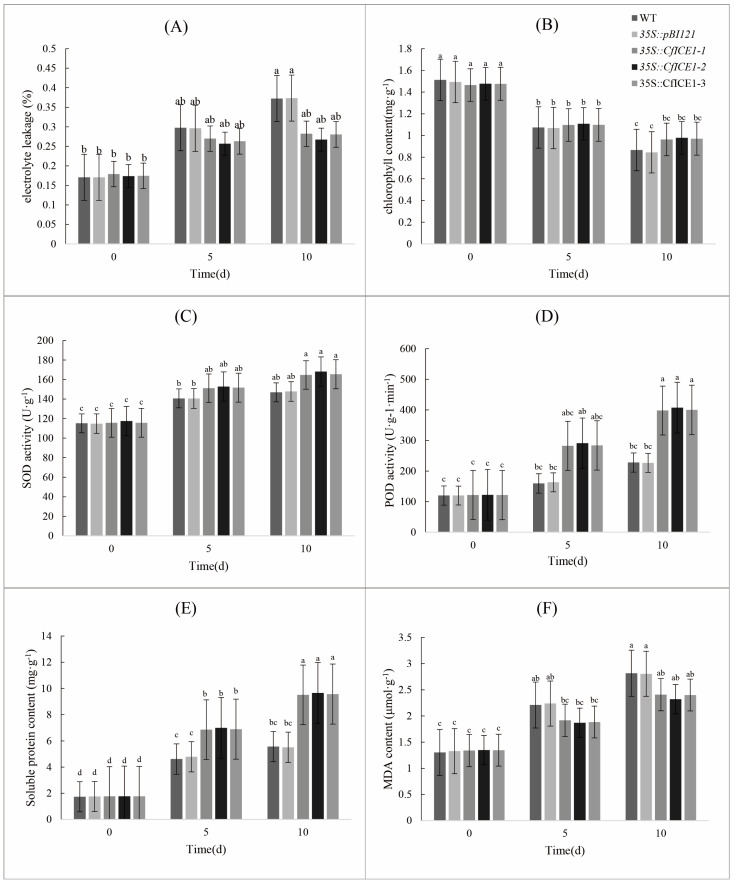
Physiological indices of transgenic, empty vector and wild-type poplar lines under drought stress: (**A**) Electrolyte leakage, (**B**) Chlorophyll content, (**C**) SOD activity, (**D**) POD activity, (**E**) Soluble protein content, (**F**) MDA content. The abscissas of all the above physiological indicators are the days of drought treatment. Each bar indicates the average ± SD (n = 3), and lowercase letters above each bar indicate significant differences (*p* ≤ 0.05).

To explore whether *CfICE1* also worked under drought stress, we treated the plants with water supply cessation. During the drought stress process, the leaves of WT and *35S::pBI121* were slightly yellowed after 5 days of drought treatment (Figure 6B), and the leaves were significantly yellowed after 10 days of drought treatment (Figure 6C). However, the transgenic plants still showed green leaves. Compared to untreated plants before drought treatment, the EL of drought stressed WT and *35S::pBI121* increased significantly, and the EL of *35S::CfICE1-1*, *35S::CfICE1-2* and *35S::CfICE1-3* did not increase significantly, even after 10 days of drought treatment (Figure 7A), suggesting that the cell membranes of the transgenic plants were more stable. After drought treatment for 5 days compared to 10 days, the chlorophyll content of WT and *35S::pBI121* was significantly different, but the chlorophyll content of transgenic plants did not change much during this period (Figure 7B). Plants treated with drought for 10 days did not suffer as much damage as those treated with salt for 8 days, as enzyme activities (SOD and POD) were increased in all five lines (Figure 7C,D). Compared with that before drought treatment, the soluble protein and MDA contents of all plants increased after treatment. Under the same treatment, the soluble protein content of transgenic plants was higher than that of WT plants (Figure 7E), and the MDA content was lower than that of WT plants (Figure 7F), which was conducive to better adaptation of the transgenic plants to stress.

### 2.5. Ultrastructure of Poplar Leaves under Different Stresses

**Figure 8:**
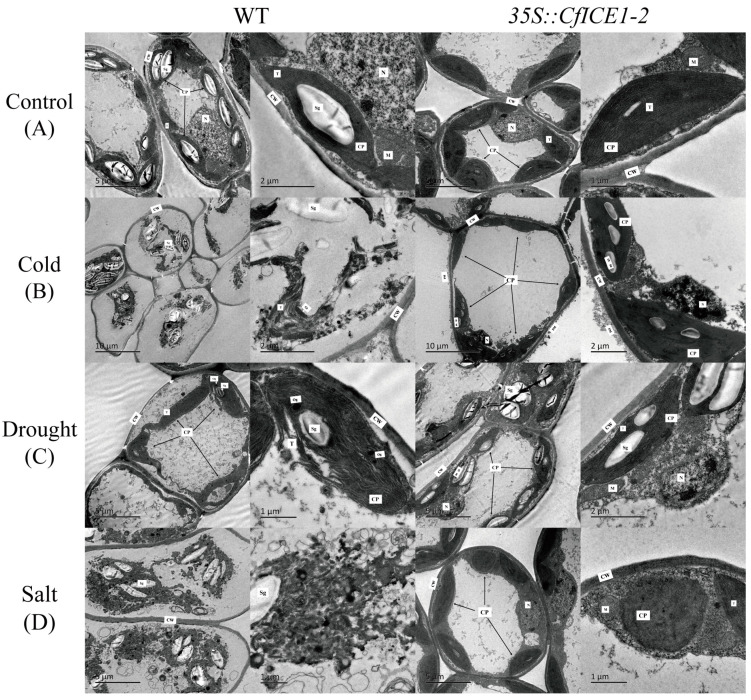
Ultrastructure of leaves of *35S:: CfICE1-2* and WT poplar lines under different stress conditions. CW: cell wall; PM: plasma membrane; N: nucleus; CP: chloroplast; M: mitochondrion; Sg: starch grain; T: thylakoid slice layer; Gt: granum-thylakoids.

By analyzing the physiological index data of transgenic, empty vector and WT poplar under cold, drought and salt stress, we observed the ultrastructure of WT and *35S::CfICE1-2* under cold stress (−8 °C), drought stress for 10 days and salt stress for 8 days. The ultrastructure of WT and transgenic leaves grown at room temperature is shown in Figure 8A. The cell wall structure of WT leaf cells in the control group was complete and clear, the internal organelles adhered to the wall, the structures of the nucleus, mitochondria and chloroplasts were complete and clear, the chloroplasts were mostly spindle shaped, the outer membrane was complete, and a clear lamellar structure could be seen. However, the chloroplasts in the transgenic leaf cells from the control group did not have large starch grains, and clear stacking areas could be seen. Under cold treatment (Figure 8B), chloroplasts in WT leaf cells were disrupted, the granum-thylakoids and thylakoid layers could be clearly seen, and the damaged organelles in some cells accumulated in the center of the cell; the ultrastructure of transgenic leaf cells under cold treatment was still relatively complete. Figure 8C shows the ultrastructure of WT and transgenic leaves after drought treatment. Chloroplasts in the WT leaf cells were far away from the cell wall and moved to the inside of the cells. No nucleus was observed, some chloroplasts collapsed, and both the chloroplast membrane and thylakoid lamella ruptured. In addition, compared with the control group, starch grains disappeared. However, starch granules appeared in chloroplasts of transgenic leaf cells after drought treatment, and some chloroplasts moved slightly to the interior of cells, but most of them were still distributed near the cell wall; some chloroplasts swelled, and nuclei were visible. Under salt treatment, the internal structure of WT leaf cells was compromised, most chloroplasts were degraded, and it was difficult to identify various organelles. The organelles in the cells of transgenic leaves were still clearly visible, some chloroplasts were swollen, the chloroplasts were deformed from spindle to irregular oval shapes, the internal structure was unclear, and the thylakoid lamellae could not be identified (Figure 8D). This was consistent with other research results: thylakoid swelling is a typical form of damage to chloroplasts under salt stress [21].

### 2.6. Analysis of DAB Staining and H_2_O_2_ Content in Poplar Leaves under Different Stresses

**Figure 9:**
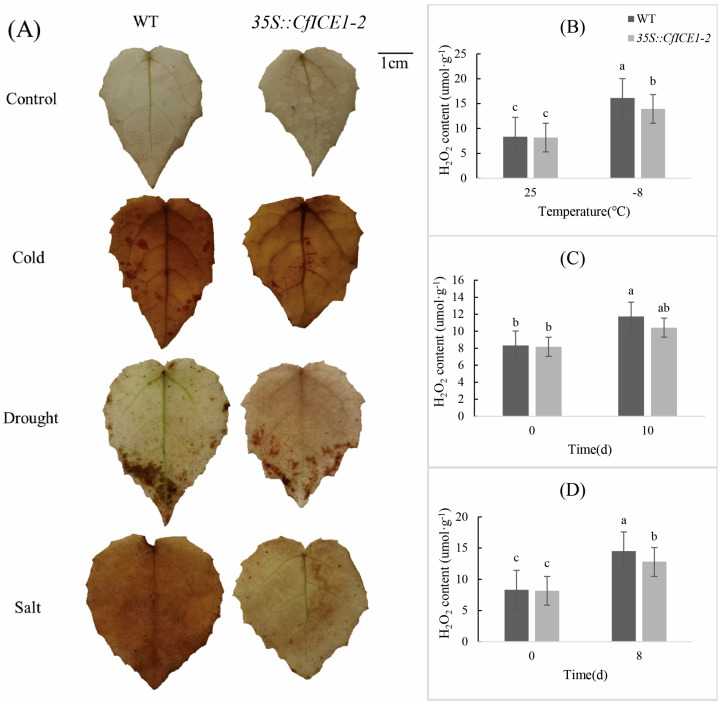
DAB staining and H_2_O_2_ content of *35S::CfICE1-2* and WT poplar lines under various stresses. (**A**) DAB staining of *35S::CfICE1-2* and WT poplar lines under cold, drought and salt stress. (**B**) H_2_O_2_ content of *35S::CfICE1-2* and WT poplar lines under cold stress. (**C**) H_2_O_2_ content of *35S::CfICE1-2* and WT poplar lines under drought stress. (**D**) H_2_O_2_ content of *35S::CfICE1-2* and WT poplar lines under salt stress. Each bar indicates the average ± SD (n = 3), and lowercase letters above each bar indicate significant differences (*p* ≤ 0.05).

Similarly, we selected WT and *35S::CfICE1-2* plants after cold stress (−8 °C), drought stress for 10 days and salt stress for 8 days for DAB staining observation (Figure 9A). The DAB staining method (diaminobenzidine method) was used to detect the active site of peroxidase in cells. Under normal conditions, the DAB staining color of *CfICE1*-overexpressing transgenic and WT leaves was similar, but after stress, the staining degree of WT leaves was different from that of *35S::CfICE1-2* (Figure 9A). After cold treatment, the plants suffered severe freezing damage, and the dyed poplar leaves were obviously dark brown, while the leaves of *35S::CfICE1-2* were lighter in color than those of WT; the H_2_O_2_ content measurements also confirmed this phenomenon (Figure 9B). After 10 days of drought stress, the whole leaves of WT were covered with brown spots, which were more obvious at the leaf edge, while *35S::CfICE1-2* only had brown spots on the leaf edge. The phenotypic observations after salt stress for 8 days showed that WT plants began wilting (Figure 4); correspondingly, the difference in leaf staining between WT and *35S::CfICE1-2* was obvious, and the accumulation of H_2_O_2_ in WT was higher than that in *35S::CfICE1-2* (Figure 9D). After plants were stressed, the content of H_2_O_2_ in plants increased rapidly, which caused oxidative stress damage. However, *CfICE1* overexpression in transgenic poplar reduced the H_2_O_2_ content and the degree of plant damage.

### 2.7. Expression of CfICE1 and PdbCBF in WT and CfICE1-Overexpressing Poplar under Different Stresses

**Figure 10:**
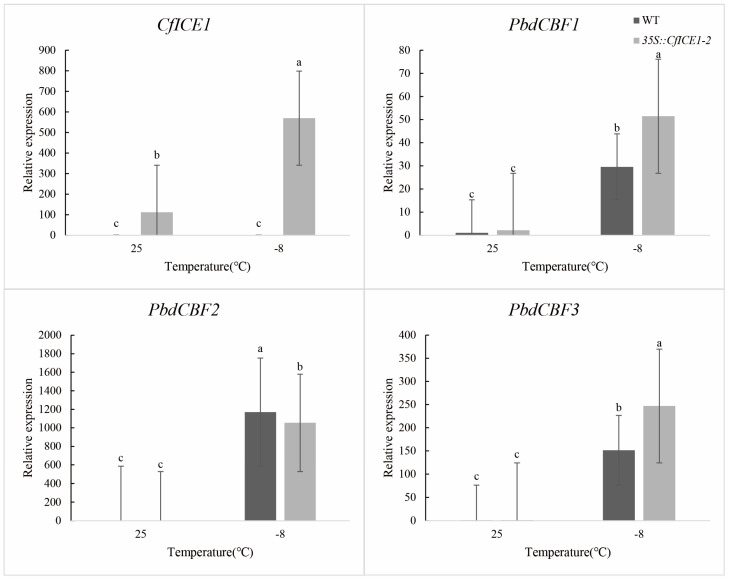
Changes in *CfICE1*, *PdbCBF1*, *PdbCBF2* and *PdbCBF3* gene expression under cold stress. Each bar indicates the average ± SD (n = 3), and lowercase letters above each bar indicate significant differences (*p* ≤ 0.05).

**Figure 11:**
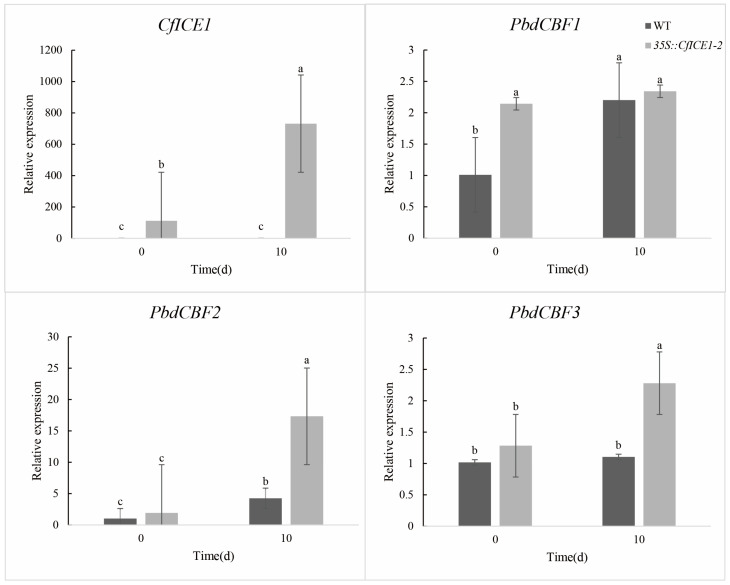
Changes in *CfICE1*, *PdbCBF1*, *PdbCBF2* and *PdbCBF3* gene expression under drought stress. Each bar indicates the average ± SD (n = 3), and lowercase letters above each bar indicate significant differences (*p* ≤ 0.05).

**Figure 12:**
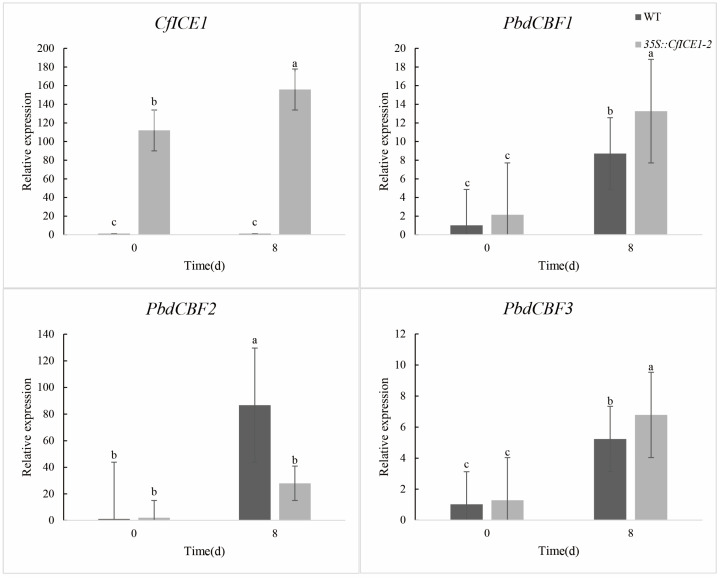
Changes in *CfICE1*, *PdbCBF1*, *PdbCBF2* and *PdbCBF3* gene expression under salt stress. Each bar indicates the average ± SD (n = 3), and lowercase letters above each bar indicate significant differences (*p* ≤ 0.05).

To better understand the regulatory role of *CfICE1* under different stresses in transgenic poplar, we first measured the change in *CfICE1* expression under cold, drought and salt stress by RT-PCR. We found that the transcriptional level of *CfICE1* was upregulated under cold, drought and salt stress (Figure 10, Figure 11 and Figure 12). To further explore whether *CfICE1* overexpression can respond to stress by affecting the expression of the *CBF* gene in poplar, we also determined the gene expression levels of *PdbCBF1*, *PdbCBF2* and *PdbCBF3* in *CfICE1*-overexpressing transgenic poplars under cold, drought and salt stress by RT-PCR (Figure 10, Figure 11 and Figure 12). We found that after cold and salt stress, the expression of *PdbCBF1* and *PdbCBF3* genes in *35S::CfICE1-2* was higher than that in WT, while the expression of *PdbCBF2* gene in *35S:: CfICE1-2* was lower than that in WT. Different from cold and salt stress, after drought stress, the expression of *PdbCBF1* in *35S:: CfICE1-2* was not significantly higher than that in WT, and the expression of *PdbCBF2* gene in *35S:: CfICE1-2* was higher than that in WT.

## 3. Discussion

Inducer of *CBF* expression 1 (*ICE1*) is a well-characterized basic helix–loop–helix (bHLH) protein, and numerous studies have shown that plant bHLH proteins are closely related to a variety of biological processes, such as the development and growth of plant reproductive organs [22], the development of root hairs and epidermal hairs [23], and the synthesis and metabolism of plant anthocyanins [24,25]. Furthermore, increasing evidence suggests that bHLH plays a key role in the response of plants to various abiotic stresses, such as cold stress [26,27], drought stress [28,29], salt stress [30,31] and iron deficiency [32]. *CfICE1* has been shown to be involved in freezing resistance of *C. fortunei*. Similar to the results of a cold resistance analysis of *CfICE1* transgenic *Arabidopsis thaliana* in a previous study from our laboratory, overexpression of *CfICE1* in transgenic poplar also helped plants better cope with cold stress. In addition, in terms of biological processes, this study found that overexpression of *CfICE1* in poplar slightly affected the formation of adventitious roots. In terms of the plant response to abiotic stress, this study found that overexpression of *CfICE1* could also improve the salt and drought tolerance of poplar.

When plants are exposed to stress conditions, including cold, drought and salt, their cellular activities and biochemical pathways change. The excessive synthesis of reactive oxygen species (ROS) causes oxidative stress in plants and leads to plant death [33]. The production of ROS triggers the activity of antioxidant enzymes in plants [34]; these enzymes can, effectively, eliminate the accumulation of ROS, thus enhancing the ability of plants to tolerate stress [35,36]. In this study, the activities of SOD and POD were increased in both WT and transgenic poplars after cold, drought and salt stresses (Figure 3, Figure 5 and Figure 7), and the antioxidant levels of *CfICE1*-overexpressing transgenic plants were superior to those of WT under each stress treatment. This suggests that the *CfICE1* gene could involve oxidative damage in plants under cold, drought and salt stress. In addition, the antioxidant enzyme activities of WT after 8 days of salt stress were significantly lower than those after 4 days of salt stress, which might be due to the inactivation of antioxidant enzymes in plants caused by severe salt stress. However, the antioxidant enzyme activities of transgenic poplars still increased after 8 days of salt stress; thus, the transgenic poplars still maintained a good phenotype (Figure 4C), while the WT leaves wilted due to their weakened ability to scavenge ROS.

H_2_O_2_ is an ROS that causes oxidative damage to plants. The accumulation of H_2_O_2_ in poplar leaves was reflected by DAB staining [37]. Compared with the WT, the *CfICE1*-overexpressing transgenic plants had less cell damage under cold, drought and salt stresses, which was proven by the DAB staining results (Figure 9A). When grown under normal conditions, the levels of H_2_O_2_ in the leaves of WT and transgenic plants were very low, and the DAB-stained area was extremely small; however, the DAB-stained areas of the leaves of transgenic and WT plants were more obvious after cold, drought or salt treatment. The staining depth of the leaves of WT was deeper than that of transgenic plants, indicating that the H_2_O_2_ content of WT was higher to that of transgenic plants. The results showed that *CfICE1* overexpression in transgenic lines increased the oxidative capacity of plants, thereby reducing the levels of oxidative damage. The level of oxidant concentration was used to determine the degree of lipid peroxidation [38], and lower oxidant levels indicated that the transgenic lines were more resistant to cold, drought and salt stress [39]. In addition, the MDA content was used to evaluate the level of lipid peroxidation, which further reflected the degree of oxidative damage to the cell membrane [40,41]. The degree of cell membrane damage could also be reflected by the amount of EL [42]. In the present study, the leakage of electrolytes in plants increased after cold, drought and salt stress, indicating that the cell membrane system was damaged. Under the same treatment, the EL and MDA content of transgenic lines were lower than those of the WT, which indicated that *CfICE1* overexpression in the transgenic lines reduced the degree of cell membrane damage. Plants usually accumulate soluble proteins after being stressed, which plays a key role in the response of plants to various abiotic stresses. This was mainly due to the strong hydrophilicity of soluble proteins, which could significantly increase the hydrophilicity, bound water content and protoplasmic elasticity of cells, thereby protecting the living substances and biofilms of cells [43]. In this study, the soluble protein content of the leaves of the *CfICE1*-overexpressing transgenic poplars was higher than that of the WT under cold, drought and salt stress.

Under the same treatment, by observing the ultrastructure of WT and *35S::CfICE1-2* leaf cells, it was found that overexpression of the *ICE1* gene in transgenic lines could better maintain the structure of intracellular organelles and better maintain cell morphology to cope with environmental stress (Figure 8). Especially under salt and cold treatment, the ultrastructural difference between WT and *35S::CfICE1-2* leaf cells was particularly obvious. Liu et al. [44] studied the chloroplast membrane localization transporter *OsNHAD* in rice, indicating that plants responded to stress by maintaining chloroplast morphology. Therefore, the chloroplast morphology in the leaves of transgenic plants after stress is more intact, and the chlorophyll content is less degraded.

In recent years, the introduction of exogenous *ICE1* to improve the tolerance of plants to cold stress has become a popular research topic; in particular, activation of the *ICE–CBF–COR* cold response pathway has enabled plants to acquire cold resistance [45]. Among the *ICE* genes studied, *ICE1* was found to be an upstream signal that directly regulates *CBF* expression. *CBF* transcription factors could specifically bind to DRE/CRT cis-acting elements, and DRE/CRT cis-acting elements were commonly found in the promoters of stress-responsive genes for drought, salt or cold stress [46]. Previous studies have shown that *CBF* can protect plants from injury by activating the expression of a large subset of downstream *COR* genes under cold and salt conditions [15]. To determine whether the *CfICE1* transgenic poplar also improved plant resistance by regulating the *CBF* gene under various stresses, we detected the expression of *ICE* downstream genes, such as *CBF1*, *CBF2* and *CBF3* after cold, drought and salt stress treatments. After cold and salt stress treatment, we found that the expression of *PdbCBF1* and *PdbCBF3* in transgenic lines was higher than that of WT. The expression of *PdbCBF2* in transgenic lines was lower than that in WT. This phenomenon under cold and salt stress supports previous findings that *CBF* can improve plant tolerance to abiotic stresses [14], the expression of *CBF1* and *CBF3* promotes the expression of *CBF2*, and *CBF2* is a negative regulator of the expression of *CBF1* and *CBF3* [47]. In this study, the expression of poplar *CBF* after drought stress was different from the above theory, that was, the expression of *PdbCBF2* in *35S::CfICE1-2* was significantly higher than that of WT. However, this phenomenon was similar to the research results of Wang et al. By studying the regulation role of poplar *PsnICE1*, Wang et al. [48] found that the expression of *PsnCBF2* in transgenic lines after treatment was higher than that of WT. In addition, there was no significant difference in the expression of *PdbCBF1* in *35S::CfICE1-2* before or after drought treatment; similarly, there was no significant difference in the expression of *PdbCBF3* in WT. This may be since drought stress had not reached the significant stage of gene expression or in the process of plant respond to drought stress, *CBF* was regulated by other genes except *ICE1* gene, and there were other more complex regulatory networks in drought resistance, which requires further research in the future. There is no denying that with the increase in stress time, the difference of some physiological indicators in transgenic and WT gradually became significant. The cell structure of transgenic poplar after drought treatment was more complete than that of WT, indicating that transgenic lines possessed better tolerance to stress compared with WT, even in severe stress.

## 4. Materials and Methods

### 4.1. Plant Materials and Growth Conditions

The materials were selected from tissue culture seedlings of poplar (*Populus davidiana×P. bolleana*) grown in a culture room (25 °C, 16 h light/8 h dark light) by our research group. After the wild-type (WT) and transgenic poplar seedlings grew to a certain height in the bottle, they were transplanted to the soil basin (peat soil:vermiculite:perlite = 2:1:1) and transferred to the controlled environment room (23 °C, 16 h light/8 h dark light) for growth.

### 4.2. DNA Extraction, RNA Extraction and cDNA Synthesis

The samples were placed in a mortar containing liquid nitrogen and quickly ground to a powder that was used to extract DNA or RNA from plants. Genomic DNA was extracted using a Super Plant Genomic DNA Kit (Polysacchardes and Polyphenolics-rich) (TIANGEN, Beijing, China). Total RNA was extracted using a Universal Plant Total RNA Extraction Kit (Spin-column) (Bioteke Corporation, Beijing, China). The RNA concentration was measured using a spectrophotometer (NanoDrop, 2000; Thermo Scientific, Waltham, MA, USA). RNA degradation and contamination were monitored by 1% agarose gel electrophoresis. The first strand of cDNA was synthesized by reverse transcription with the HiScript III 1st Strand cDNA Synthesis Kit (Vazyme Biotechnology Co., Ltd., Nanjing, Jiangsu, China) and used for gene cloning. The cDNA was synthesized by reverse transcription with HiScript III RT SuperMix for qPCR (Vazyme) and used as a template for qPCR analysis.

### 4.3. Genetic Transformation in Poplar

The ORF of *CfICE1* was amplified using primers with restriction sites (Table S1). We used a CBI Express^®^ II One Step Cloning Kit C112 (Vazyme) to ligate the target fragment into the PBI121 vector that was double digested by BamHI and XbaI. Then, the recombinant product was transferred into *Escherichia coli* DH5α (Vazyme) for sequencing. The recombinant expression vector plasmid that showed the correct sequencing results was transferred into *Agrobacterium tumefaciens* strain EHA105 (Shanghai Weidi Biotechnology Co., Ltd., Shanghai, China) for genetic transformation.

*Agrobacterium* containing the target gene was transferred into poplar by the leaf disc method [49,50], after which the infected poplar leaves were cultured on MS medium containing 50 mg/L kanamycin and 200 mg/L cefotaxime to induce transgenic plants. The DNA of antibiotic-resistant plants was extracted as a template and detected by PCR to remove false-positive plants, and further qPCR experiments were performed to identify plants with high expression levels.

### 4.4. Physiological Index Analysis of Plants Treated with Cold, Salt and Drought

WT and transgenic poplars grown in a controlled environment chamber were collected for stress treatment. Poplar trees were placed in an ultralow temperature freezer for low-temperature treatment. The experimental group was treated at −4 °C, −8 °C, −12 °C, and −16 °C for 12 h, and the control group was treated at room temperature. After treatment, the samples were allowed to slowly return to room temperature, and index measurements were performed. In the salt tolerance experiment, the plants were treated with 300 mmol/L NaCl aqueous solution for 4 and 8 consecutive days. In the drought stress experiments, the plants were first given sufficient water, followed by water supply cessation and continued drought treatment for 4 and 8 days. The control group was watered normally.

Chlorophyll content was determined by spectrophotometry. The samples were extracted by grinding with quartz sand, calcium carbonate and 95% ethanol and were centrifuged to obtain a supernatant. The final optical density (OD) values of the supernatants were determined at multiple wavelengths (649 nm and 665 nm) using a spectrophotometer, and a formula [51] was used to calculate the chlorophyll concentration. Fresh leaves were collected and soaked in distilled water, and after standing in the laboratory for 12 h, a conductivity meter was used to measure the conductivity (c1) of the aqueous solution. Then, the aqueous solution containing the leaves was boiled in water for 15 min, and the conductivity (c2) of the aqueous solution was measured with the same method after cooling. Finally, EL was calculated according to the formula (c1/c2 × 100%). SOD, POD, MDA and soluble protein contents were determined using azalantetrazole (NBT) colorimetry, guaiacol oxidation, the thiobarbituric acid (TBA) method and Coomassie brilliant blue G-250 staining, respectively [52,53].

### 4.5. Transmission Electron Microscopy (TEM) Analysis

Leaves of WT and transgenic poplars were sampled after cold, drought and salt treatments. WT and transgenic poplars, which were normally watered and held at room temperature, were used as controls. Fresh leaves were collected, and the leaf portions outside the main vein were cut into rectangles of approximately 2 × 1 mm. The leaves were placed in a 5 mL penicillin bottle with 2 mL of glutaraldehyde solution, and the air in the bottle was drawn with a syringe so that the leaves completely sank to the bottom of the bottle. Samples could be temporarily stored in a 4 °C refrigerator. In short, the leaves were fixed, rinsed, dehydrated, embedded, sliced and double stained before being viewed and photographed under a JEM-1400 transmission electron microscope.

### 4.6. DAB Staining and Determination of the H_2_O_2_ Content

The leaves of WT and transgenic poplars were sampled at room temperature and after different stress treatments. The washed and dried leaves were completely soaked in a light-protected bottle containing DAB staining solution, after which they were placed on a shaker at 25 °C 80 r/min for overnight staining. After staining, the leaves were transferred to a bottle containing 95% ethanol, which was placed in boiling water, and after the chlorophyll in the leaves had completely faded, the depigmented leaves were photographed. To quantitatively describe the degree of plant damage, we used the titanium sulfate colorimetric method to detect the hydrogen peroxide (H_2_O_2_) content of poplar leaves. Acetone was used to disrupt cells, homogenize tissue, and dilute liquid samples; then, we added 0.2 mL of concentrated ammonia water and 0.1 mL of 5% titanium sulfate to 1 mL of the liquid sample. The yellow precipitate produced by the reaction was dissolved in 5 mL of 2 mol/L H_2_SO_4_, and the maximum absorption peak was measured by colorimetry at a wavelength of 410 nm [54]. The OD values were substituted into the standard curve prepared in advance to calculate the H_2_O_2_ concentration. Within a certain range, the depth of color in the liquid after the precipitate dissolved was linearly related to the H_2_O_2_ concentration.

### 4.7. Semiquantitative PCR and Real-Time Quantification PCR

The cDNA samples of poplar collected from the experimental group and the control group were used as templates for quantitative PCR analysis. Multiple pairs of primers for target genes were designed on the NCBI Primer-BLAST website (https://www.ncbi.nlm.nih.gov/tools/primer-blast/ (accessed on 27 October 2022)), and the best pair of primers (Table S1) was selected by semiquantitative analysis for subsequent real-time quantification. The poplar genes UBQ and RG1 were used as reference genes. Real-time quantitative PCR was performed according to the method of Zhang et al. [19], and the data were checked using 7500 software v2.3 (Applied Biosystems, Waltham, MA, USA). Among them, 96-Well 0.2ml Semi Skirt PCR Plates and other experimental consumables were obtained from NEST Biotechnology Co. Ltd. (Wuxi, China).

### 4.8. Statistical Analysis

All experiments were performed with at least three replicates for each line/treatment. The statistical analysis and analysis of variance were performed using Microsoft Excel 2019 and IBM SPSS 26.0, respectively. The level of significance was set to *p* < 0.05. The graphs were processed using Adobe Illustrator CS6.

## 5. Conclusions

In this study, we further explored the role of *CfICE1* in perennial woody plants. We found that *CfICE1* not only plays an active regulatory role in enhancing cold stress but also enhances the salt tolerance and drought resistance of perennial woody plants. In addition, *CfICE1* overexpression also affected the expression of *CBF1*, *CBF2* and *CBF3* in poplar itself under cold and salt stress, which further verified the existence of the *ICE–CBF* pathway in plants. The current research expands our understanding of the function and potential mechanisms of *CfICE1*.

## Figures and Tables

**Table 1:** Statistics of the plant height, leaf length, width and length/width in wild-type and transgenic poplars. Values shown are mean ± SD (n = 9); *p* values of Student’s test for transgenic lines and controls poplars are denoted with asterisks.

Types	WT	*35S::pBI121*	*35S::CfICE1-1*	*35S::CfICE1-2*	*35S::CfICE1-3*
Height (cm)	15.22 ± 0.91	14.56 ± 0.42	11.61 ± 0.55 **	13.61 ± 0.55 *	11.67 ± 0.85 **
Length (cm)	4.39 ± 0.31	3.90 ± 0.45	3.51 ± 0.36	3.78 ± 0.47	3.54 ± 0.41
Width (cm)	3.57 ± 0.06	3.03 ± 0.57	2.79 ± 0.38	3.02 ± 0.55	2.80 ± 0.38
Length/Width	1.23 ± 0.07	1.31 ± 0.13	1.26 ± 0.04	1.26 ± 0.08	1.27 ± 0.1

*, *p* < 0.05; **, *p* < 0.01.

## Data Availability

Not applicable.

## Supplementary Materials

The following supporting information can be downloaded at: https://www.mdpi.com/article/10.3390/ijms232315214/s1.

## Abbreviations

*ICE1* ,	inducer of *CBF* expression 1;
*CBF*,	C-repeat binding factor;
*COR*,	cold-regulated gene;
bHLH,	basic helix–loop–helix;
WT,	wild-type;
ORF,	open reading frame;
OD,	optical density;
EL,	electrolyte leakage;
DAB,	diaminobenzidine;
H_2_O_2_,	hydrogen peroxide;
SOD,	superoxide dismutase;
POD,	peroxidase;
MDA,	malondialdehyde;
ROS,	reactive oxygen species.
