# Supplementary material for: Overexpression of CfICE1 from Cryptomeria fortunei Enhances Cold, Drought and Salt Stress in Poplar
Source: Int J Mol Sci. 2022 Dec 2;23(23):15214. doi: 10.3390/ijms232315214 (PMC9736380; doi:10.3390/ijms232315214)

Table S1. Primers used in this study.

Primer name	Primer sequence (5'-3')	Application
XbaI-CfICE1-F	gagaacacgggggactctagaATGTTCTCGAGAATGAACACTGGA	Amplification of the conserved region
BamHI-CfICE1-R	ggactgaccacccggggatccCATTGCAGAATGACAGCTTGCA	
RT-*CfICE1*-F	TGCTCTTTAGGCCCAAGCAA	RT-PCR
RT-*CfICE1*-R	GGCACCCACCGAAGGATAAA	
UBQ-F	GTTGATTTTTGCTGGGAAGC	
UBQ-R	GATCTTGGCCTTCACGTTGT	
RG1-F	AAACATTGGACGGGGTGCTC	
RG1-R	CCCACATGAGGCTGGAGGAC	
PbdCBF1-F	CGGATTCTGCTTGGAGGTTG	
PbdCBF1-R	TCAACTCACCTCCCTCACAC	
PbdCBF2-F	TCGGGTAAGTGGGTTTGTGA	
PbdCBF2-R	AGCAACATCATGTGCCCTTG	
PbdCBF3-F	GGGCGGAGGATATTCAAGGA	
PbdCBF3-R	GGATACGTGCCTAGCCAGAT	

Figure S1. Identification of transgenic plants by qRT-PCR. The figure shows the measured expression of each plant after treatment at 4 °C. According to the gene expression level and plant growth status, transgenic poplar 2 (*35S::CfICE1-1*), transgenic poplar 3(*35S::CfICE1-2*) and transgenic poplar 4 (*35S::CfICE1-3*) were finally selected to complete the experiment. Each bar indicates the average ± SD (n = 3), and lowercase letters above each bar indicate significant differences (p ≤ 0.05).

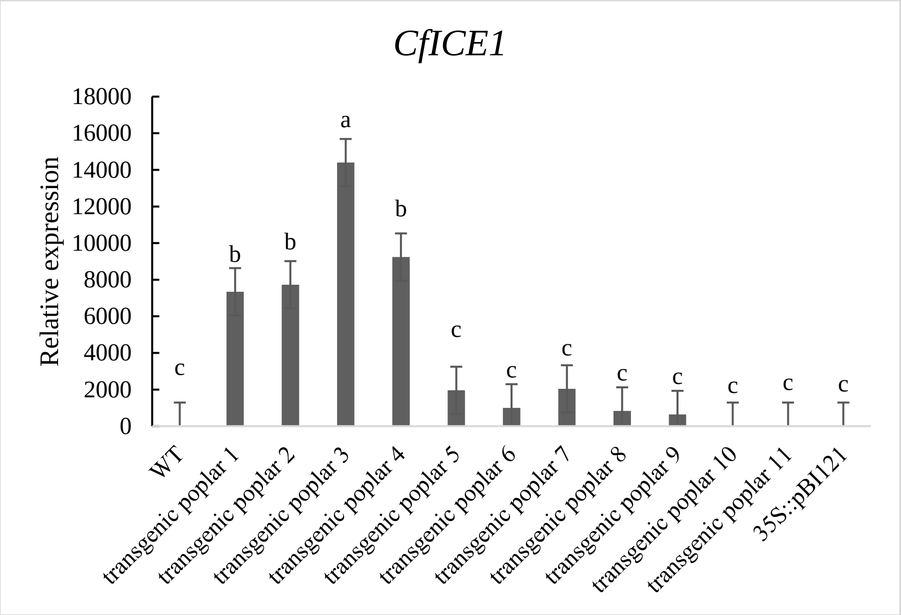

Supplement: Supplementary file 1 [file ijms-23-15214-s001.zip › ijms-2028816-supplementary.pdf]
